# Color Distribution Differences in the Tongue in Sleep Disorder

**DOI:** 10.1155/2014/323645

**Published:** 2014-04-28

**Authors:** Chang Jin Jung, Ji Ho Nam, Young Ju Jeon, Keun Ho Kim

**Affiliations:** Medical Engineering R&D Group, Korea Institute of Oriental Medicine, 461-24 Jeonmin-dong, Yuseong-gu, Daejeon 305-811, Republic of Korea

## Abstract

*Introduction*. According to traditional East Asian medicine (TEAM) theory, the tongue represents conditions of qi and blood. In the present study, the relationship between the tongue and the qi and blood in conditions with no apparent disease was investigated. *Methods*. A total of 454 elderly people with no apparent disease were recruited. Two Korean oriental medicine doctors classified subjects into a normal group (*n = 402*) and a sleep disorder group (*n = 52*). Three to five weeks after the experiment, 153 subjects were rerecruited for a second experiment. Two-dimensional color histograms, whose seven variables represent the color distribution in Commission Internationale de l'Éclairage 1976 (*L*∗, *a*∗, *b*∗) color space, were produced from tongue images. *Results*. The color of the tongue body in the sleep disorder group appeared paler than that in the normal group, and the tongue coating in the normal group was less widely distributed compared with that in the sleep disorder group. The differences in tongue color between the normal at first experiment and sleep disorder at second experiment conditions were similar to the differences between the normal and the sleep disorder groups. *Conclusions*. The tongue states in the sleep disorder group indicate a qi and blood deficiency according to TEAM theory.

## 1. Introduction

Traditional East Asian medicine (TEAM) posits that the tongue reflects pathological conditions and functional states of organs [1]. The tongue provides direct evidence for the diagnosing of a patient's syndrome based on its visual information, and it has been frequently used in TEAM clinics. The tongue body changes to a paler, reddish, or purplish color according to the symptom profiles of patients, and the tongue coating is related to the functional state of the stomach qi and internal organs [2].

The tongue exhibits different states during the progression of a syndrome. The state of the qi and blood, which is an essential concept in TEAM, is related to the tongue from the early stages of a syndrome to the final stages. A deficiency of the qi and blood at an early stage of a syndrome is represented in the tongue [1]. Tongue manifestations are advantageous for the assessment of health conditions with or without apparent disease according to the TEAM theory of associations between the tongue and the state of qi and blood.

Although the tongue represents useful information for diagnosis, a physiological mechanism associated with tongue color and coating has not been clearly established. Current clinical approaches for investigating the relationship between the tongue and disease have used digital tongue images and demonstrated clinical meanings of tongue manifestations [3–7]. The digital tongue images, which are acquired in a controlled environment, allow for the quantification of color information and the performance of an objective study to establish the physiological mechanisms of the tongue.

The tongue color of subjects with sleep disorder and no apparent disease was investigated in the present study by using digital tongue images to verify the relationship between the tongue and the qi and blood in conditions with no apparent disease. Differences in the tongues of individuals with no apparent disease were smaller than those in patients with sleep disorders in previous studies. Therefore, we proposed a color histogram method for the extraction of a detailed color distribution of the tongue and found trends of differences in sleep disorder subjects compared with subjects without a sleep disorder.

## 2. Methods

### 2.1. Study Subjects and Experimental Procedures

**Figure 1:**
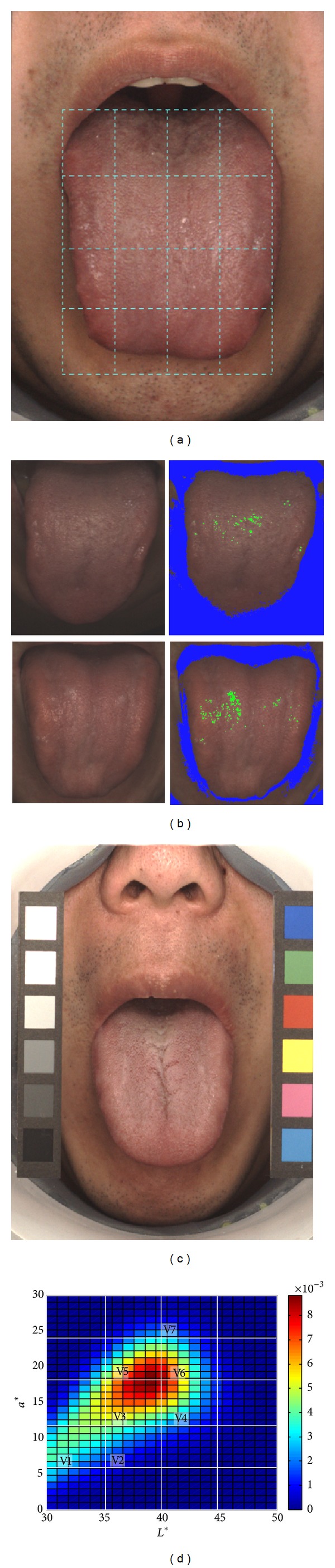
Methods of color image analysis. (a) Example of the feedback guidelines on the monitor, which are shown to a subject during the image acquisition process. (b) Examples of the dark and high-luminance regions in the acquired images. Green pixels indicate high-luminance regions with L* values over 85, and blue pixels indicate dark regions with L* values under 30. (c) A color checker in the tongue image for the color correction analysis. The left 6 samples and right 6 samples were used for achromatic and chromatic color correction, respectively. (d) Average of the two-dimensional color histogram of 454 subjects. Seven color ranges (V1–V7) were selected for statistical analysis.

From June 2012 to April 2013, 454 elderly people with no apparent disease were recruited from the Cheonan Oriental Hospital of Daejeon University. Two Korean oriental medicine doctors independently interviewed subjects about current sleep problems to assess whether they had a sleep disorder. The sleep disorder group was defined as subjects with a sleep disorder, based on agreement between the two Korean oriental medicine doctors (*n* = 52), and subjects with no sleep disorder were classified into the normal group (*n* = 402). A tongue image was acquired after the diagnostic process using a tongue diagnosis system (TDS), which provides feedback during the image acquisition process and a color checker for color correction, as shown in Figures 1(a) and 1(c) [3]. The subjects abstained from eating and drinking for 2 hours before the experiment to prevent color changes of the tongue body and coating. Three to five weeks after the experiment, 153 subjects in the normal group were rerecruited for a second experiment to observe tongue color changes according to the presence of a sleep disorder in the same subject. Eighteen of the 153 subjects showed a sleep disorder change, and these subjects were used for a paired comparison analysis.

### 2.2. Image Processing

Pixel values in the red-green-blue (RGB) color space from the acquired images were converted to the Commission Internationale de l'Éclairage (CIE) 1976 (L*, a*, b*) color space, which is device-independent. The CIE 1976 (L*, a*, b*) color space includes all human-perceivable colors and consists of L*, a*, and b* coordinates. Positive values of a* indicate red or magenta, and positive values of b* indicate yellow. Negative values of a* and b* indicate green and blue, respectively. The L* values represent color lightness. The tongue color and its change are well represented by the a* value because the color of a normal tongue is pale red, and it becomes paler, blue, or purplish according to the subject's pathological conditions.

A tongue region was automatically segmented by using the combined polar edge method [8] and the gradient vector flow snake technique [9]. For a missegmented contour, a modification process that included manual and automatic segmentation was repetitively performed. A dark region of the tongue root area, which was caused by low illumination intensity, and a high-luminance region, which was caused by light reflection from saliva, were removed by using thresholding with the L* values because these colors could introduce error into the color histogram analysis. The threshold values of the dark and high-luminance regions were defined as 30 and 85, respectively, as shown in Figure 1(b), and these thresholds were applied to the whole tongue images. Color correction was performed in the CIE 1976 (L*, a*, b*) color space to reduce the color distortion due to acquisition devices and the influence of unexpected external light sources. For the color correction, 12 color samples of the color checker in the acquired tongue image were used (Figure 1(c)), and 3 linear equations for each L*, a*, and b* coordinate were solved by using the least squares method. Six achromatic color samples were used for L* value corrections, and 6 chromatic color samples were used for a* and b* value corrections.

### 2.3. Color Histogram Analysis

A two-dimensional color histogram (TDCH) was proposed for the extraction of detailed color information because it is possible that the color differences of the tongue in the sleep disorder group could be smaller than those in patients with apparent disease. The TDCH counted the number of pixels in each color range based on the L* and a* coordinates and represented the L* and a* distribution of the tongue color. The L* and a* domains of the TDCH were derived from the mean color distribution of the 454 subjects. Intervals of the L* and a* domains were determined as 5 and 6, respectively, which divide each coordinate into 4 ranges. Small domain intervals of less than 5 could not be determined because of acquisition errors associated with tongue color in the present experiment. Sixteen color ranges for the histogram analysis were defined from four criteria values (L*: 30, 35, 40, and 45; a*: 6, 12, 18, and 24) in two-dimensional space. The number of pixels was counted and normalized for each color range by dividing the number of pixels in each color range by the number of pixels in the entire tongue area. The normalized values represent the proportion of the area with pixels within the color range.

Among the 16 histogram ranges, ranges with rarely distributed color in the tongue were excluded from the analysis. Based on the TDCH values of the 454 subjects, seven TDCH variables were selected, as shown in Figure 1(d). V1 indicated the proportion of area with a dark and pale red color, and V7 indicated the proportion of area with a bright and reddish color.

### 2.4. Statistical Analysis

Differences in the seven TDCH variables between the normal (*n* = 402) and the sleep disorder (*n* = 52) groups were calculated. A normality test was performed for each variable before the calculation using the Kolmogorov-Smirnov and Shapiro-Wilk tests [10]. The seven variables showed nonnormal distributions, and a Mann-Whitney *U* test was performed for the group difference analyses [11].

Changes in the TDCH variables according to the presence of sleep disorder from the first and second experiments were calculated in 18 subjects. All variables showed normal distributions according to the Kolmogorov-Smirnov and Shapiro-Wilk tests, and a one-sample Student's *t*-test was used for a paired comparison analysis between the normal at first experiment (NAFE) and the sleep disorder at second experiment (SASE) subjects.

## 3. Results

### 3.1. Differences in the Tongue Color between the Normal and the Sleep Disorder Groups

Differences in the TDCH variables between the normal and the sleep disorder groups were assessed in the first experiment. The demographics of each group are shown in Table 1. The mean age of the two groups was 57 years, and no differences in diastolic blood pressure, pulse, or body temperature were observed between the two groups. The mean value of systolic blood pressure in the sleep disorder group tended to be lower than that in the normal group (*P* = 0.051). The normality of each variable was tested, and all variables revealed nonnormal distributions, as shown in Table 2.

**Figure 2:**
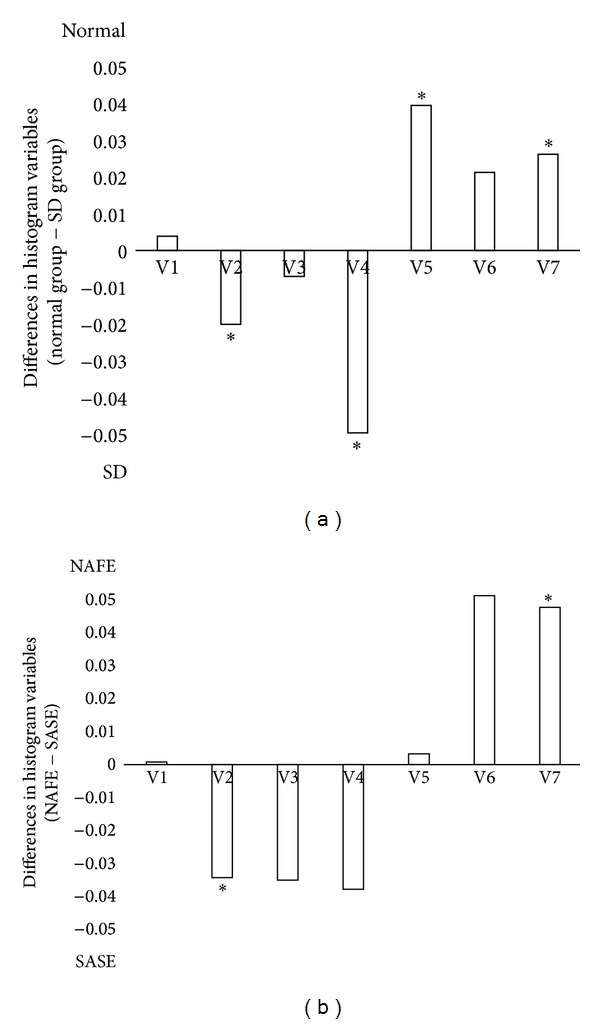
Differences in the histogram variables between normal and sleep disorder (SD) subjects. (a) Variable differences between the normal (*n* = 402) and SD (*n* = 52) groups in the first experiment, *P < 0.05. (b) Variable differences in the same subjects (*n* = 18) who were normal in the first experiment (NAFE) and had SD in the second experiment (SASE), *P < 0.05.

Medians and interquartile variable ranges in the two groups are shown in Table 3. V2, V4, V5, and V7 showed significant differences between the two groups according to the Mann-Whitney *U* test (*P* < 0.05). The median values of V2 and V4 in the sleep disorder group were higher than those in the normal group. In contrast, the median values of V5 and V7 in the sleep disorder group were smaller than those in the normal group. In both groups, the median values of V6 were larger than those of other variables, but this difference was not significant (*P* = 0.61). V4 and V5 were significantly different between groups, as shown in Figure 2(a).

### 3.2. Differences in Tongue Color between the Same Subjects with and without a Sleep Disorder

Eighteen subjects in the normal group for the first experiment showed SASE. The normality of variables in these 18 subjects in the two experiments was tested, and all variables exhibited normal distributions (Table 4). Differences in the variables between the NAFE and SASE conditions were calculated, and V2 and V7 showed significant differences in a two-sample paired Student's *t*-test, as shown in Table 5 (*P* < 0.05). V4 tended to be lower in the NAFE than the SASE, but this difference was not significant (*P* = 0.55). The results of the paired comparison analysis were similar to those of the group difference analysis of the first experiment, except V5, as shown in Figure 2. V6 was the largest of the seven variables in the NAFE condition, but V3 was the largest in the SASE condition. V2, V3, and V4 in the SASE condition tended to be larger than those in the NAFE condition, and V6 and V7 in the SASE condition tended to be smaller than those in the NAFE condition. Four variables showed trends of differences between the NAFE and SASE conditions after the exclusion of V1, V3, and V5.

## 4. Discussion

**Figure 3:**
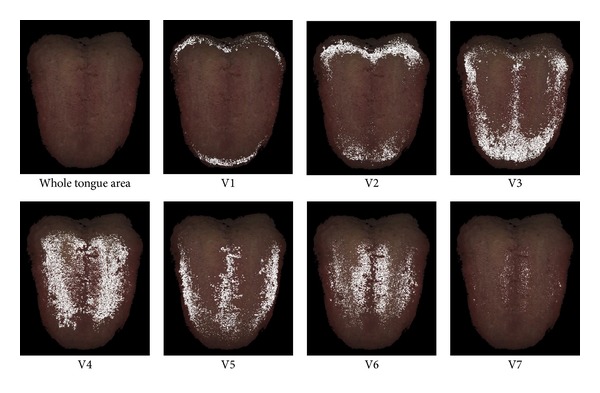
Example of areas corresponding to the color ranges of the seven variables in a tongue image. The areas were described using white pixels in the white tongue image. A dark region in the root of the tongue and a light reflected area were excluded from the analysis.

The color properties of the tongue image depend on the state of the tongue body and the coating. The tongue coating is a fur-like substance that covers the surface of the tongue, and its color differs from the color of the tongue body. The seven variables of the TDCH included the color ranges of both the tongue body and the tongue coating because the color ranges were derived from the color distribution of the entire tongue area. The results of the difference analyses between the two groups revealed that V2, V3, and V4 with low a* ranges tended to contrast with V5, V6, and V7 with high a* ranges. The contrasting trends of these variables imply that there are two differences in the tongue state between the normal and the sleep disorder groups. The first difference is the proportion of the tongue coating area. The a* dimension of the TDCH, which describes the intensity of the red color, is one of the crucial parameters of the tongue body and coating [12].  Figure 3 shows areas corresponding to the color ranges of the seven variables in a tongue image. The seven areas in Figure 3 indicate that V1, V2, and V4 were related to the proportion of the tongue coating area, and V5, V6, and V7, whose a* ranges were higher than those of the other variables, represent the tongue body area. The trends of the relationships between the V1, V2, and V4 and their corresponding tongue areas appeared similar in all subjects because the optical properties between the tongue surface and substance of the tongue coating were different. The values of V2 and V4 were larger in the sleep disorder group than in the normal group, which indicates that the area proportion of the tongue coating in the sleep disorder group was higher than that in the normal group. The values of V5 and V7, which represent the tongue body, in the sleep disorder group were smaller than those in the normal group because of the high proportion of the tongue coating area. The second difference in the tongue state between the two groups was the color of the tongue body.  Figure 3 shows that three color ranges of V3, V5, and V7 were observed in the tongue body. The a* and L* ranges of V7 were the highest of the seven variables, and the a* range of V3 was lower than that of V5 and V7. V3 appeared to be related to the pale red color of the tongue body based on the red intensity, whereas V7 appeared to be related to a bright and intense red tongue body. The ratio between the V3 and V7 values depended on the degree of the red intensity of the tongue body. The V7 value was larger in the normal group than in the sleep disorder group.  Table 6 shows the difference in the V7-to-V3 ratio (V7/V3) between the two groups. The ratio in the normal group was higher than that in the sleep disorder group, which suggests that the red intensity of the tongue body in the normal group was higher than that in the sleep disorder group. The color of the tongue body in the sleep disorder group appeared paler than that in the normal group, and the proportion of tongue coating area in the sleep disorder group was higher than that in normal group.

Elderly people with no apparent disease participated in these experiments, and the subjects were classified as having sleep disorder based on interviews assessing the subjects' sleep disturbances. A variety of issues, including psychological problems (e.g., anxiety, depression, and stress [13, 14]), and painful physical conditions [15, 16], may cause sleep disorders. Research on the elderly has shown that sleep disorder is related to chronic disease, such as heart disease, lung disease, and osteoporosis [17]. Since variable issues contribute to sleep disorders, common physiological states in the sleep disorder group were more related to physiological influences derived from the sleep disorder rather than to the issues, which cause the sleep disorder. Fatigue is a frequently observed symptom in sleep disorders due to the insufficient sleep duration. Previous studies reported that sleep disorders, which are caused by various factors, are related to the symptom of fatigue [18–22]. The physiological states of the subjects in the sleep disorder group are likely primarily related to fatigue.

The color of the tongue body and the tongue coating indicate pathological conditions of the body independently of each other in TEAM. The color of the tongue body is generally pale red, and it becomes paler, more intense red, blue, or purple according to the symptom profiles of individual patients [23]. The tongue color in the sleep disorder group was paler than that in the normal group in this study. According to TEAM theory, a pale tongue is observed in patients with a qi or blood deficiency. A pale tongue with a blood deficiency in patients with fatigue can be interpreted in conjunction with the blood state. The tongue receives its blood supply primarily from the lingual artery, which is a branch of the external carotid artery [24]. The dorsal surface of the tongue is composed of a mucous membrane and covered in lingual papillae. The pale red color of the tongue surface in a normal state comes from a combination of colors of the mucous membrane (CMMs), which may reflect the optical properties of the blood (OPB) in the lingual artery and the lingual papillae. A reddish and shiny tongue surface color is observed in atrophic glossitis, which is caused by the loss of lingual papillae [25]. Various studies have shown that the hematological parameters in subjects with fatigue, especially chronic fatigue (CF), differ from those in healthy subjects. The red blood cell (RBC) distribution and RBC magnesium levels appear decreased in patients with CF [26, 27], and cardiac output and blood volume, including plasma and RBC volume, in CF patients are lower than those in healthy subjects [28]. The trend of systolic blood pressure difference between the two groups in the results was associated with the low cardiac output and blood volume in the fatigue. The abnormality in RBC distribution and RBC magnesium in the CF, which are related to the hemoglobin and RBC counts [29], respectively, provide evidence for the OPB and CMM differences in subjects with fatigue. A noninvasive prediction method for the quantification of RBCs using spectroscopy of the tongue surface has been proposed [30], and it showed evidence of the relationship between the CMM of the tongue and OPB. The pale tongue in the sleep disorder group likely represents the physiological state of fatigue and decreased RBC counts, and it shows the relationship between the tongue body color and the state of the blood.

The tongue coating is related to the state of stomach qi and the conditions of internal organ function in TEAM [31, 32]. The tongue surface of a patient with a functional decline in the stomach or stomach qi deficiency is wildly covered by the tongue coating. The tongue coating in the sleep disorder group was more widely distributed compared with that in the normal group. The tongue coating in the sleep disorder group appeared related to the functional decline of the stomach, but the mechanism associated with the correlation between the tongue coating, fatigue, and stomach function has not been clearly established. Metabolites in chronic gastritis and chronic hepatitis B patients are related to the tongue coating [33, 34]. The tongue coating appears to be associated with metabolism and stomach function, and intensive research is required to establish the physiological meaning of the tongue coating.

Our results revealed that the trends of the differences between the normal and the sleep disorder groups were similar between the NAFE and the SASE conditions. The differences between the NAFE and the SASE conditions indicate that the color of the tongue body becomes paler and the proportion of the tongue coating increases in the sleep disorder condition. The changes in the SASE condition were related to the physiological condition during the short-term phase rather than the chronic phase because the first and the second experiments were performed at intervals of 3 to 5 weeks. We observed that sleep disorders changed the states of the tongue body and coating to tongue conditions typical of fatigue, and the tongue states reflected the manifestation of qi deficiency and blood deficiency, which are associated with fatigue. These results suggest a relationship between the tongue and the state of qi and blood under conditions of no apparent disease.

Table 7 shows the classification results using the seven TDCH variables in the first experiment based on Naïve Bayes, *k*-nearest neighbor, and support vector machine methods [35, 36]. The 454 subjects were classified into the two groups with classification accuracy over 85% in the Naïve Bayes method. The high accuracy means that the seven TDCH variables represent the differences of the tongue condition in the sleep disorder and the state of qi and blood under the conditions of no apparent disease. The TDCH analysis of the tongue image is believed to be helpful in the diagnosis of deficiency syndromes in the clinic.

## 5. Conclusions

Differences in the tongue color between the normal and the sleep disorder groups were derived from the seven TDCH variables in this study. The color of the tongue body in the sleep disorder group appeared paler than that in the normal group, and the tongue coating in the normal group was less widely distributed compared with the distribution in the sleep disorder group. The tongue states in the sleep disorder group showed typical conditions of the qi and blood deficiency syndrome according to TEAM, which represents the physiological states of fatigue. The differences in the tongue in the sleep disorder group imply that the tongue represents the state of qi and blood during conditions of no apparent disease. It is expected that the seven TDCH variables derived from the tongue image will aid in the diagnosis of deficiency syndromes in conjunction with fatigue in the clinic.

## Figures and Tables

**Table 1:** Demographics of the normal and sleep disorder groups.

Characteristics	Normal	Sleep disorder	*P* value
Number of samples	402	52	
Age (mean ± SD)	57.425 ± 5.475	57.019 ± 5.641	0.616
Height (cm) (mean ± SD)	158.020 ± 6.975	157.283 ± 5.705	0.936
Weight (kg) (mean ± SD)	60.494 ± 9.192	60.387 ± 7.575	0.465
SBP (mmHg) (mean ± SD)	72.828 ± 9.723	70.942 ± 9.166	0.051
DBP (mmHg) (mean ± SD)	119.483 ± 16.454	114.750 ± 15.934	0.186
Pulse (mean ± SD)	70.460 ± 9.678	71.692 ± 8.910	0.384
Body Temp. (mean ± SD)	36.408 ± 0.233	36.342 ± 0.584	0.132

SD: standard deviation, SBP: systolic blood pressure, DBP: diastolic blood pressure, and Body Temp.: body temperature; *P* values for variables were derived from the two-sample Student's *t*-test.

**Table 2:** Results of the normality test of the color histogram variables in the normal and sleep disorder groups.

Group	Kolmogorov-Smirnov			Shapiro-Wilk		
	Statistic	d.f	*P* value	Statistic	d.f	*P* value
V1						
Normal	0.104	402	0.000	0.901	402	0.000
SD	0.089	52	0.200*	0.979	52	0.476
V2						
Normal	0.208	402	0.000	0.691	402	0.000
SD	0.141	52	0.011	0.905	52	0.001
V3						
Normal	0.103	402	0.000	0.924	402	0.000
SD	0.186	52	0.000	0.921	52	0.002
V4						
Normal	0.115	402	0.000	0.926	402	0.000
SD	0.139	52	0.013	0.923	52	0.002
V5						
Normal	0.044	402	0.059	0.978	402	0.000
SD	0.143	52	0.010	0.885	52	0.000
V6						
Normal	0.037	402	0.200*	0.992	402	0.038
SD	0.080	52	0.200*	0.977	52	0.395
V7						
Normal	0.113	402	0.000	0.916	402	0.000
SD	0.221	52	0.000	0.816	52	0.000

V1–V7: color histogram variables, SD: sleep disorder, and d.f: degrees of freedom, *a lower bound of the true significance in Lilliefors significance correction.

**Table 3:** Color histogram variables in the normal and sleep disorder groups.

Variables	Normal (*n* = 402)	SD (*n* = 52)	*P* value
V1	^†^0.048 (0.033)	0.048 (0.038)	0.798
V2	0.042 (0.047)	0.062 (0.092)	0.048*
V3	0.130 (0.094)	0.127 (0.103)	0.577
V4	0.080 (0.097)	0.136 (0.168)	0.002*
V5	0.130 (0.104)	0.075 (0.088)	0.000*
V6	0.211 (0.088)	0.185 (0.122)	0.061
V7	0.082 (0.122)	0.038 (0.116)	0.008*

SD: sleep disorder, ^†^median (interquartile range); *P* values for variables were derived from the Mann-Whitney *U* test (*P < 0.05).

**Table 4:** Results of the normality test of the color histogram variables in the paired comparison analysis.

Variables	Kolmogorov-Smirnov			Shapiro-Wilk		
	Statistic	d.f	*P* value	Statistic	d.f	*P* value
V1	0.095	18	0.200*	0.986	18	0.990
V2	0.109	18	0.200*	0.959	18	0.590
V3	0.099	18	0.200*	0.985	18	0.989
V4	0.125	18	0.200*	0.966	18	0.728
V5	0.097	18	0.200*	0.961	18	0.621
V6	0.181	18	0.124	0.957	18	0.548
V7	0.190	18	0.084	0.946	18	0.359

d.f: degrees of freedom, *a lower bound of the true significance in Lilliefors significance correction.

**Table 5:** Paired comparison of the color histogram variables in the same subjects between the first and second experiments.

Variables	Normal (*n* = 18)	SD (*n* = 18)	*P* value
V1	^†^0.050 (0.035)	0.049 (0.021)	0.938
V2	0.108 (0.064)	0.133 (0.061)	0.019*
V3	0.172 (0.066)	0.198 (0.086)	0.135
V4	0.145 (0.074)	0.173 (0.085)	0.055
V5	0.077 (0.069)	0.074 (0.079)	0.087
V6	0.207 (0.085)	0.169 (0.081)	0.075
V7	0.086 (0.076)	0.050 (0.072)	0.047*

Normal: subjects who were normal in the first experiment; SD: subjects with sleep disorder in the second experiment, ^†^mean (standard deviation); *P* values for variables were derived from the one-sample Student's *t*-test (*P < 0.05).

**Table 6:** Difference in V7-to-V3 ratios according to the presence of sleep disorder.

	Normal (*n* = 402)	Sleep disorder (*n* = 52)	*P* value
VR	^†^0.574 (1.334)	0.267 (1.133)	0.019*

VR: V7-to-V3 ratios (V7/V3) in the normal and sleep disorder groups in the first experiment, ^†^median (interquartile range); *P* values for variables were derived from the Mann-Whitney *U* test (*P < 0.05).

**Table 7:** Classification results using the seven TDCH variables in the first experiment.

	Naïve Bayes	KNN	SVM
Accuracy	85.24%	81.72%	72.47%

KNN: *k*-nearest neighbor; SVM: support vector machine; 10-fold cross-validation was used for evaluating accuracies of the three classification methods.
